# Nomogram prediction for cervical lymph node metastasis in multifocal papillary thyroid microcarcinoma

**DOI:** 10.3389/fendo.2023.1140360

**Published:** 2023-05-26

**Authors:** Wen-Hui Li, Wei-Ying Yu, Jia-Rui Du, Deng-Ke Teng, Yuan-Qiang Lin, Guo-Qing Sui, Hui Wang

**Affiliations:** Department of Ultrasound, China-Japan Union Hospital of Jilin University, Changchun, Jilin, China

**Keywords:** nomogram, ultrasound radiomics, multifocal, papillary thyroid microcarcinoma, cervical lymph node metastasis

## Abstract

**Aim:**

Accurate preoperative prediction of cervical lymph node metastasis (LNM) in patients with mPTMC provides a basis for surgical decision making and the extent of tumor resection. This study aimed to develop and validate an ultrasound radiomics nomogram for the preoperative assessment of LN status.

**Methods:**

A total of 450 patients pathologically diagnosed with mPTMC were enrolled, including 348 patients in the modeling group and 102 patients in the validation group. Univariate and multivariate logistic regression analyses were performed on the basic information, ultrasound characteristics, and American College of Radiology Thyroid Imaging Reporting and Data System (ACR TI-RADS) scores of the patients in the modeling group to identify independent risk factors for LNM in mPTMC and to construct a logistic regression equation and nomogram to predict the risk of LNM. The validation group data were used to evaluate the predictive performance of the nomogram.

**Results:**

Male sex, age <40 years, a single lesion with a maximum diameter >0.5 cm, capsular invasion, a maximum ACR score >9 points, and a total ACR score >19 points were independent risk factors for the development of cervical LNM in mPTMC. Both the area under the curve (AUC) and concordance index (C-index) of the prediction model constructed from the above six factors were 0.838. The calibration curve of the nomogram was close to the ideal diagonal line. Furthermore, decision curve analysis (DCA) demonstrated a significantly greater net benefit of the model. The external validation demonstrated the reliability of the prediction nomogram.

**Conclusions:**

The presented radiomics nomogram, which is based on ACR TI-RADS scores, shows favorable predictive value for the preoperative assessment of LNs in patients with mPTMC. These findings may provide a basis for surgical decision making and the extent of tumor resection.

## 1 Introduction

According to the World Health Organization classification, papillary thyroid microcarcinoma (PTMC) is defined as papillary thyroid cancer (PTC) with a maximum diameter ≤1 cm. PTMC with ≥2 nodules is defined as multifocal papillary thyroid microcarcinoma (mPTMC), which accounts for 20-50% of PTMC.

Some studies have shown that the risk of lymph node metastasis (LNM), locoregional recurrence (LRR) or distant metastases is higher for mPTMC than for unifocal PTMC (uPTMC) (1–3), and some studies of low-risk mPTMC did not show clinical progression after long-term active surveillance (4). mPTMC has a wide variation in prognosis; therefore, its treatment is controversial (5, 6). Some studies have suggested that prophylactic central neck dissection (PCND) with total thyroidectomy (TT) is a significantly more efficient method to reduce the risk of LRR (7–10). However, some studies do not support the routine use of PCND in the treatment of patients with cN0 PTC10 because PCND + TT increased the incidence rate of temporary and permanent hypoparathyroidism and temporary laryngeal nerve injury (LNI) (5).

The 2015 American Thyroid Association (ATA) Practice Guidelines recommend that active monitoring can be implemented instead of surgical treatment for patients with low-risk mPTMC, while more aggressive surgical treatment should be adopted for patients with high-risk mPTMC (11). Cervical LNM is an important criterion for judging the risk of mPTMC and an important indicator for assessing the invasiveness and prognosis of mPTMC (1, 12).

Confronted with a disease such as mPTMC, which is highly controversial in terms of treatment modalities, physicians should focus on ways to better predict the natural history of disease (13). LNM is a breakthrough point in predicting disease regression. mPTMC with combined LNM is more aggressive than mPTMC without LNM.

LNM can be detected by auxiliary examinations, such as ultrasound and computed tomography (CT). However, due to the complexity of the neck structure, gas interference, and the small metastatic lymph node volume, the detection accuracy and sensitivity are low, and establishing a basis for clinical treatment is difficult (2, 14, 15).

In this study, mPTMC served as the study object. Univariate and multivariate logistic regression analyses were performed on the basic information, ultrasound characteristics, and American College of Radiology Thyroid Imaging Reporting and Data System (ACR TI-RADS) (16) scores of patients to Screen independent risk factors for cervical LNM in mPTMC patients. Nomograms are widely used for cancer prognosis, primarily because of their ability to reduce statistical predictive models into a single numerical estimate of the probability of an event, such as death or recurrence. Our study constructs a nomogram to predict cervical LNM in mPTMC patients through multivariate analysis results, providing guidance for clinical decision-making.

## 2 Materials and methods

### 2.1 Research subjects

This study used a single-center retrospective design, and data from all participating patients were anonymous. Therefore, this study was approved by the Research Ethics Committee, and the requirement for informed consent was waived.

Medical records from January 2019 to December 2019 were retrieved from the database of our hospital. A total of 450 eligible patients were enrolled, 348 of which were included in the modeling group, while 102 were included in the validation group.

Inclusion criteria: (1) preoperative ultrasound examination showing ≥2 suspected malignant nodules with postoperative pathological confirmation of mPTMC; (2) total thyroidectomy was performed in our hospital with central lymph node dissection (CLND) and/or lateral lymph node dissection (LLND); (3) postoperative pathological confirmation of the presence or absence of LNM; (4) no other treatment for thyroid diseases before surgery; (5) preoperative ultrasound examination results with complete images for each lesion meeting the assessment requirements; and (6) no history of head and neck radiation exposure.

Exclusion criteria: (1) a postoperative pathological type other than mPTMC or other types of thyroid cancer; (2) clinical and/or pathological detection of distant metastasis; (3) the presence of other malignant tumors; and (4) a family history of thyroid cancer or a history of other head and neck diseases.

### 2.2 Instruments and methods

The ultrasound examinations of all patients were independently performed by two physicians with more than 10 years of experience. A Mindray Resona 8 US unit (Mindray, China) with an L14-5 WU linear probe was used. The final diagnosis was established through consultation between the two physicians when discrepancies occurred.

Each patient was placed in the supine position, with the neck fully exposed, and thyroidectomy was performed transversely and longitudinally to avoid missing nodules. Suspicious nodules were examined by transverse and longitudinal section scanning.

**Figure 1:**
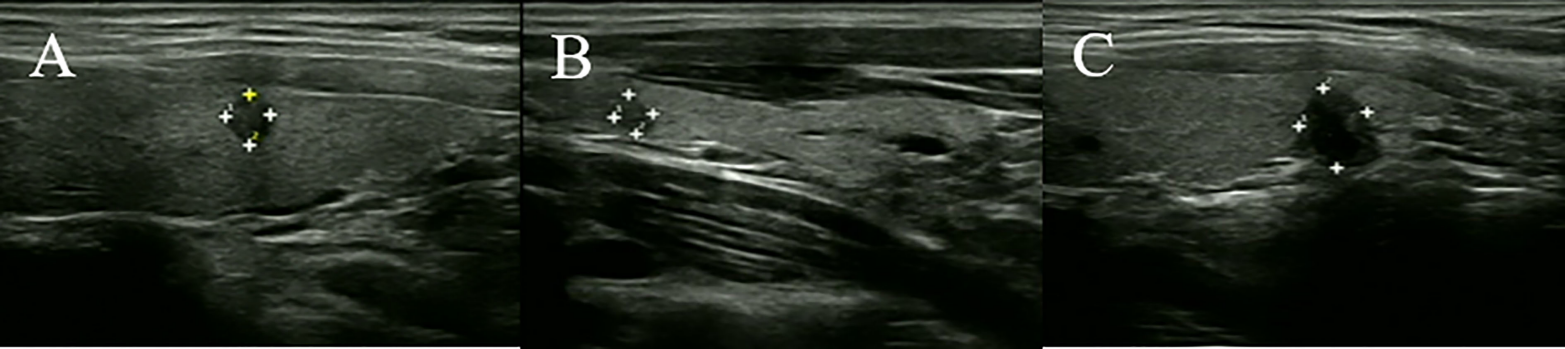
Ultrasound images of case X. **(A)** Nodule 1. **(B)** Nodule 2. **(C)** Nodule 3.

In the modeling group, according to postoperative pathology and ACR TI-RADS, we recorded the following information of the PTMC which was diagnosed by postoperative pathology for each case: gender, age, number of PTMC nodules, number of thyroid lobes occupied by PTMCs, largest diameter of each PTMC, largest diameter of the case, TTD of all PTMCs, thyroid capsule invasion of each PTMC, highest ACR score and Total ACR scores and TDR. For example, the Case X is A 43-year-old male who has three nodules in both thyroid lobes. Postoperative pathology confirms that they are all PTMC. The preoperative ultrasound findings are as follows: nodule 1 (**Figure 1A**): Very hypoechoic echogenicity in the middle and lower left lobe, 0.40cm × 0.46cm in size, taller-than-wide in shape, irregular in margin and punctate echogenic foci is visible inside; nodule 2 (**Figure 1B**): Very hypoechoic echogenicity in the middle and lower left lobe, 0.62cm × 0.84cm in size (inner side adjacent to trachea), taller-than-wide in shape, irregular in margin and punctate echogenic foci visible inside; nodule 3 (**Figure 1C**): Very hypoechoic echogenicity at the upper pole of the right lobe, with a size of 0.34cm × 0.40cm, taller-than-wide in shape, irregular in margin and punctate echogenic foci visible inside.

The information recording process for this case is as follows: Male, 43 years old, with 3 lesions and 2 thyroid lobes were occupied by thyroid nodules. The maximum diameter of each PTMC is 0.46cm, 0.84cm, and 0.40cm, respectively. The largest diameter of this case is 0.84cm, TTD=0.46 + 0.84 + 0.40 = 1.7cm, and there is no capsule invasion. Referring to ACR TI-RADS, the ACR scores of the three PTMCs are: 13 points, 13 points, and 13 points, respectively. Therefore, the Highest ACR score is 13 points, the Total ACR score is 13 + 13 + 36 points, and TDR=13/36 = 0.33 points. Finally, we recorded the following information for this case: male, 43 years old, 3 lesions, 2 lobes, largest diameter=0.84cm, TTD=1.7cm, no capsule invasion, highest ACR score=13 points, total ACR score=36 points, TDR=0.33. Other cases in the modeling group will collect the required information through this process and proceed to the next research and analysis.

In the validation group: First, we select TR4/TR5 nodules according to the ACR TI-RADS, and then record the following information: gender, age, number of PTMC nodules, number of thyroid lobes occurred by PTMCs, largest diameter of each PTMC, largest diameter of the case, TTD of all PTMCs, thyroid capsule invasion of each PTMC, highest ACR score, and Total ACR scores and TDR. The calculation methods such as largest diameter and TTD are the same as modeling group. The validation group information was substituted into NOMO to assess the risk of LNM and compared with postoperative pathology to evaluate the predictive efficacy of NOMO.

Receiver operating characteristic (ROC) curves were used to calculate the optimal cutoff points as the grouping basis.

According to postoperative paraffin pathology, patients without LNM were recorded as negative for LNM, and patients with LNM in the central and/or lateral cervical regions were recorded as positive for LNM.

### 2.3 Statistical analysis

Statistical analyses were performed using R software 4.2.0 and SPSS 27.0 software. Qualitative variables are expressed as frequencies and composition ratios. Univariate logistic regression analysis was performed on the clinical and ultrasound characteristics and ACR TI-RADS scores of the patients in the modeling group, and significant factors (P<0.1) were selected as independent variables (χ). Multivariate binary logistic regression analysis was performed, and whether the postoperative paraffin pathological results indicated LNM was used as the dependent variable (Y) to obtain independent risk factors for LNM in mPTMC. A value of P<0.05 was considered statistically significant. Based on the identified risk factors, a nomogram of risk factors associated with LNM in mPTMC was established.

**Figure 2:**
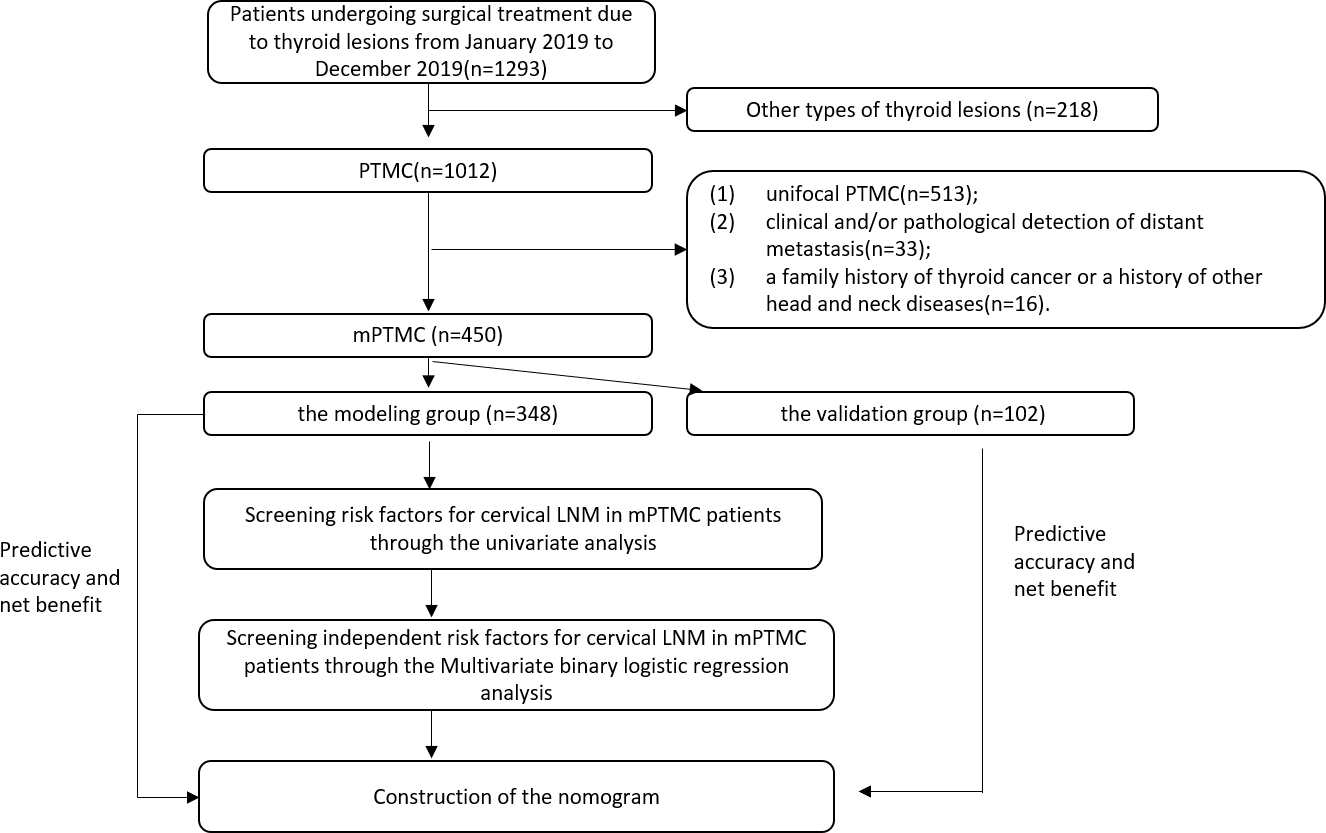
The flow chart of the whole experiment.

The ROC curve, area under the ROC curve (AUC), concordance index (C-index), and calibration curve were used to evaluate the predictive accuracy and conformity of the model. Decision curve analysis (DCA) reflected the net benefit of the model for patients (**Figure 2**).

## 3 Results

### 3.1 Patient information

This study included 450 mPTMC patients with a pathological diagnosis, including a total of 1183 lesions, with 348 patients in the modeling group (901 lesions) and 102 patients in the validation group (282 lesions). The comparisons between the modeling group and validation group are summarized in **Table 1**.

**Table 1:** Comparisons between modeling group and validation group.

Factor		Modeling group			Validation group			
		LNM+ (%)	LNM- (%)	Total number of cases (%)	LNM+ (%)	LNM- (%)	Total number of cases (%)	*p*
χ_1_: Sex	Female	113(38.2%)	183(61.8%)	296(85.1%)	28(32.6%)	58(67.4%)	86(84.3%)	0.854
	Male	36(69.2%)	16(30.8%)	52(14.9%)	9(56.3%)	7(43.8%)	16(15.7%)	
χ_2_: Age	<40 years	67(54.0%)	57(46.0%)	124(35.6%)	23(60.5%)	15(39.5%)	38(37.3%)	0.696
	40-50 years	51(33.6%)	101(66.4%)	152(43.7%)	10(25.0%)	30(75.0%)	40(39.2%)	
	≥50 years	31(43.1%)	41(59.6%)	72(20.7%)	4(16.7%)	20(83.3%)	24(23.5%)	
χ_3_: Number of thyroid nodules	2	90(39.5%)	138(60.5%)	228(65.5%)	15(27.3%)	40(72.7%)	55(53.9%)	0.033
	>2	59(49.2%)	61(50.8%)	120(34.5%)	22(46.8%)	25(53.2%)	47(46.1%)	
χ_4_: Number of thyroid lobes occupied by thyroid nodules	1	50(44.6%)	62(55.4%)	112(32.2%)	7(17.5%)	33(82.5%)	40(39.2%)	<0.001
	2	95(41.1%)	136(58.9%)	231(66.4%)	23(42.6%)	31(57.4%)	54(52.9%)	
	3	4(80.0%)	1(20.0%)	5(1.4%)	7(87.5%)	1(12.5%)	8(7.9%)	
χ_5_: Largest diameter	≤0.5 cm	43(26.7%)	118(73.3%)	161(46.3%)	9(19.6%)	37(80.4%)	46(45.1%)	0.835
	>0.5 cm	106(56.7%)	81(43.3%)	187(53.7%)	28(50.0%)	28(50.0%)	56(54.9%)	
χ_6_: TTD	≤0.8 cm	42(27.6%)	110(72.4%)	152(43.7%)	12(25.5%)	35(74.5%)	47(46.1%)	0.668
	>0.8 cm	107(54.6%)	89(45.4%)	196(56.3%)	25(45.5%)	30(54.5%)	55(53.9%)	
χ_7_: Capsular invasion	No	74(32.5%)	154(67.5%)	228(65.5%)	21(31.8%)	45(68.2%)	66(64.7%)	0.880
	Yes	75(62.5%)	45(37.5%)	120(34.5%)	16(44.4%)	20(55.6%)	36(35.3%)	
χ_8_: Highest ACR score	≤9	38(23.5%)	124(76.5%)	162(46.6%)	18(36.7%)	31(63.3%)	49(48.0%)	0.791
	>9	111(59.7%)	75(40.3%)	186(53.4%)	19(35.8%)	34(64.2%)	53(52.0%)	
χ_9_: Total ACR scores	≤19	64(31.5%)	139(68.5%)	203(58.3%)	19(31.7%)	41(68.3%)	60(58.8%)	0.930
	>19	85(58.6%)	60(41.4%)	145(41.7%)	24(57.1%)	18(42.9%)	42(41.2%)	
χ_10_: TDR	≤0.5	48(47.1%)	54(52.9%)	102(29.3%)	11(35.5%)	20(64.5%)	31(30.4%)	0.833
	>0.5	101(41.1%)	145(58.9%)	246(70.7%)	26(36.6%)	45(63.4%)	71(69.6%)	

Modeling group: The average age was 42.80 ± 9.456 years (16-68); the number of nodules ranged from 2 to 8, including 228 (65.5%) cases with 2 lesions, 68 (38%) cases with 3 lesions, 33 (9.5%) cases with 4 lesions, 10 (10%) cases with 5 lesions, 5 (1.4%) cases with 6 lesions, 3 (0.9%) cases with 7 lesions, and 1 (0.3%) case with 8 lesions; the maximum diameter range from 0.1 to 1.0cm; the average TTD was 1.0144 ± 0.47757 cm (0.2-3.3); the average Total ACR scores was 20.0 ± 8.797 points (8-67); the average TDR was 0.6162 ± 0.16813(0.13-1.00).

Validation group: The average age was 42.37 ± 9.581 years (23-68); the number of nodules ranged from 2 to 8, including 55 (53.9%) cases with 2 lesions, 24 (23.5%) cases with 3 lesions, 12 (11.8%) cases with 4 lesions, 5 (4.9%) cases with 5 lesions, 3 (2.9%) cases with 6 lesions, 2 (2.0%) cases with 7 lesions, and 1 (1.0%) case with 8 lesions; the maximum diameter range from 0.1 to 1.0cm; the average TTD was 0.9938 ± 0.46966 cm (0.2-3.3); the average Total ACR scores was 19.98 ± 9.386 points (8-61); the average TDR was 0.6087 ± 0.18346 (0.13-1.00).

We conduct a univariate analysis of the following factors: gender, age, number of PTMC nodules, number of thyroid lobes occupied by PTMCs, largest diameter of the case, TTD of all PTMCs, thyroid capsule invasion of each PTMC, highest ACR score and Total ACR scores and TDR.

### 3.2 Risk factors for cervical LNM in mPTMC patients

The univariate analysis results indicated that male sex ((χ_1_) (P<0.001), age <40 years (χ_2_) (P=0.004), the number of thyroid nodules (χ_3_) (P=0.083), largest diameter in a single case >0.5 cm (χ_5_) (P<0.001), TTD in a single case >0.8 cm (χ_6_) (P<0.001), capsular invasion (χ_7_) (P<0.001), total ACR score in a single case >19 points (χ_8_) (P<0.001) and highest ACR score in a single case >9 points (χ_9_) (P<0.001) were correlated with cervical LNM in mPTMC (P<0.1). The number of thyroid lobes occupied by thyroid nodules (χ_4_) (P=0.264) and the tumor diameter ratio (TDR) (χ_10_) (P=0.303) were not correlated with cervical LNM. The results of the univariate logistic regression analysis are shown in **Table 2**.

**Table 2:** Results of the univariate logistic regression analysis of risk factors for cervical LNM.

Risk factors	*β*	*SE*	*χ^2^*	*df*	*P*	*OR*	95%*CI*	
							Lower limit	Upper limit
χ_1_ Sex	1.293	0.323	15.985	1	<0.001	3.644	1.933	6.868
χ_2_ Age			11.519	2	0.003			
<40 years						1		
40-50 years	-0.845	0.249	11.519	1	<0.001	0.430	0.264	0.700
≥50 years	-0.441	0.299	2.185	1	0.139	0.643	0.358	1.155
χ_3_ Number of thyroid nodules	0.394	0.227	3.004	1	0.083	1.483	0.950	2.316
χ_4_ Number of thyroid lobes occupied by thyroid nodules			2.666	2	0.264			
1						1		
2	-0.144	0.232	0.382	1	0.536	0.866	0.549	1.366
3	1.601	1.134	1.994	1	0.158	4.960	0.537	45.794
χ_5_ Largest diameter >0.5cm	1.278	0.231	30.546	1	<0.001	3.591	2.282	5.651
χ_6_ TTD >0.8cm	1.147	0.231	24.599	1	<0.001	3.149	2.001	4.954
χ_7_ Capsular invasion	1.244	0.236	27.839	1	<0.001	3.468	2.185	5.505
χ_8_ Highest ACR score >9 points	1.575	0.238	43.718	1	<0.001	4.829	3.028	7.702
χ_9_ Total ACR scores >19 points	1.124	0.226	24.646	1	<0.001	3.077	1.974	4.795
χ_10_ TDR	-0.244	0.237	1.059	1	0.303	0.784	0.493	1.247

### 3.3 Independent risk factors for cervical LNM in mPTMC patients

Multivariate binary logistic regression analysis was performed on variables with significance in the univariate analysis, and the analysis results indicated that male sex (χ_1_), age <40 years (χ_2_), largest diameter >0.5 cm (χ_5_), capsular invasion (χ_7_), highest ACR score >9 points (χ_8_), and total ACR score >19 points (χ_9_) were independent risk factors for cervical LNM in mPTMC. The risk of cervical LNM in men was 3.808 times that of women (P<0.001), the risk in patients aged 40-50 years was 0.331 times that of patients aged <40 years (P<0.001), and the risk in patients aged ≥50 years was 0.451 times that of patients aged <40 years old (P=0.032), suggesting that age <40 years may be a risk factor for cervical LNM. The risk in patients whose largest tumor diameter was >0.5 cm was 4.665 times that in patients with a single lesion with a largest diameter ≤0.5 cm (P<0.001). The risk of cervical LNM in patients with capsular invasion (χ_7_) was 3.773 times that in patients without capsular invasion (P<0.001). The risk in patients whose highest ACR score >9 points was 5.497 times that of patients whose highest ACR score ≤9 points (P<0.001). The risk in patients whose total ACR score >19 points (χ_9_) was 1.916 times that in patients whose total ACR score ≤19 points (P=0.023). The multivariate logistic regression produced the following equation: Y=−2.459+1.3371−1.1072(2)−0.7972(3)+1.5405+1.3287+1.7048+0.6509 . The multivariate logistic regression analysis results are shown in **Table 3**.

**Table 3:** Results of multivariate logistic regression analysis of risk factors for cervical LNM.

Risk factors	*β*	*SE*	*χ^2^*	*df*	*P*	*OR*	*95%CI*	
							Lower limit	Upper limit
χ_1_ Sex	1.337	0.395	11.467	1	0.001	3.808	1.756	8.257
χ_2_ Age			12.791	2	0.002			
<40 years						1		
40-50 years	-1.107	0.315	12.377	1	<0.001	0.331	0.178	0.613
≥50 years	-0.797	0.371	4.614	1	0.032	0.451	0.218	0.933
χ_5_ Largest diameter>0.5cm	1.540	0.292	27.906	1	<0.001	4.665	2.634	8.261
χ_7_ Capsular invasion	1.328	0.288	21.304	1	<0.001	3.773	2.147	6.631
χ_8_ Highest ACR score>9 points	1.704	0.307	30.781	1	<0.001	5.497	3.010	10.036
χ_9_ Total ACR scores>19 points	0.650	0.286	5.155	1	0.023	1.916	1.093	3.358
Constant	-2.459	0.371	43.847	1	<0.001	0.085		

### 3.4 Construction of the nomogram

**Figure 3:**
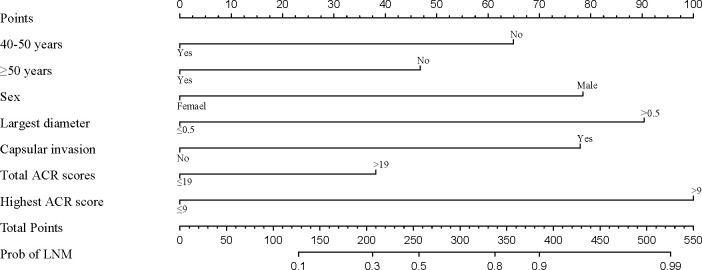
Nomogram for the prediction of LNM occurrence in mPTMC.

The nomogram was constructed based on the above six factors (R2 = 0.448, C-index=0.838) (**Figure 3**). For each patient, a greater number of total points indicated a higher risk of LNM. For example, if a 43-year-old man has three thyroid nodules, all with ACR TI-RADS scores of 13, with the largest diameter being 0.8 cm, and is negative for capsular invasion, then the corresponding scores would be approximately 46, 78, 100, 38, 90, and 0, respectively; for a total score of approximately 352, indicating an LNM risk of 83% for this patient. Postoperative pathological results suggesting LNM positivity would be consistent with the predicted results.

To use this nomogram in individual patients, the information for 7 (axes 2-8 axis) risk factors should be visualized as a point on the first axis. Then, the sum of these 7 points out of the total number of points should be plotted on axis 9. Then, a line is drawn downwards toward the risk axis (axis 10) to determine the likelihood of recurrence for an individual patient.

### 3.5 Predictive accuracy and net benefit of the nomogram

**Figure 4:**
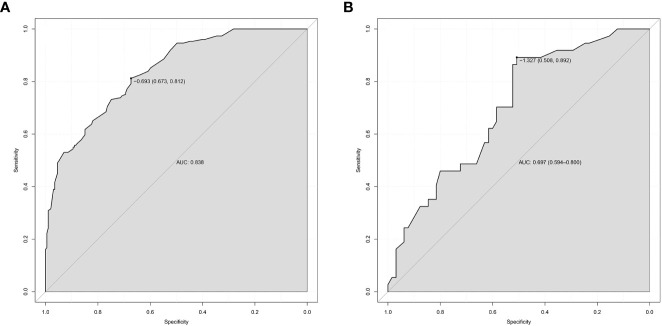
ROC curves. **(A)** Modeling group. **(B)** Validation group. ROC, receiver operating characteristic; AUC, area under the ROC curve.

**Figure 5:**
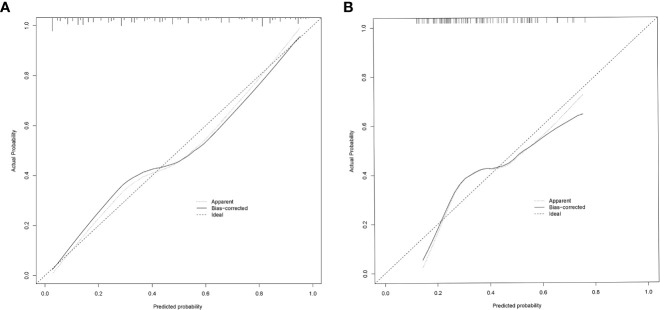
Calibration curve for predicting probability of LNM in mPTMC. **(A)** Modeling group. **(B)** Validation group; LNM, lymph node metastasis; mPTMC, multifocal papillary thyroid microcarcinoma.

**Figure 6:**
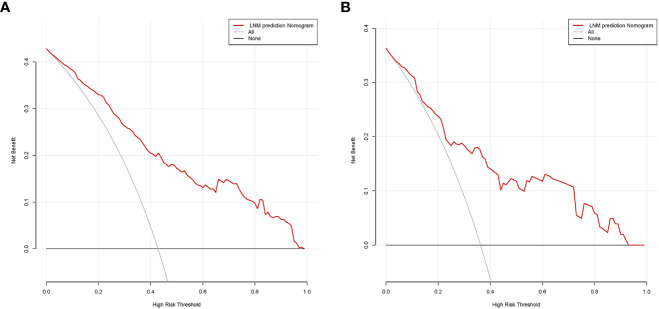
Decision curve analysis in prediction of LNM in mPTMC. **(A)** Modeling group. **(B)** Validation group. LNM, lymph node metastasis; mPTMC, multifocal papillary thyroid microcarcinoma.

In the modeling group, the AUC was 0.838 (**Figure 4A**), and the calibration curve was close to the ideal diagonal line (**Figure 5A**). Furthermore, DCA showed a significantly greater net benefit of the nomogram (**Figure 6A**). In addition, 102 patients from our hospital were used for external validation to test the nomogram. The AUC was 0.697 (**Figure 4B**), reflecting good accuracy of the nomogram. Meanwhile, the nomogram had good consistency, and the calibration curve of the validation group was also close to the ideal diagonal line (**Figure 5B**). Moreover, DCA also showed a significant net benefit of the nomogram in the validation group (**Figure 6B**). These data demonstrated that our nomogram had significant potential for clinical decision making. For example, the following results were obtained: female, 44 years old, largest diameter=0.70cm, TTD=1.1cm, no capsule invasion, highest ACR score=10 points, total ACR score=31 points. Then, the corresponding risk for LNM was 30%.

Physicians and patients can use the NOMO to predict the risk of LNM and individually assess patients more accurately to help them choose a more appropriate treatment plan.

## 4 Discussion

In our study, univariate and multivariate logistic regression analyses were performed on factors that may be associated with the development of LNM in mPTMC. The analysis results indicated that male sex, age <40 years, largest diameter >0.5 cm, capsular invasion, highest ACR score >9 points, and total ACR score >19 points are independent risk factors for cervical LNM in mPTMC.

Previous studies on predictive models for the development of LNM in PTMC did not discuss uPTMC versus mPTMC separately, but their analyses also showed that male sex, age <40 years, largest diameter >0.5 cm, and capsular invasion were independent risk factors for the development of LNM in PTMC, which is consistent with the results of this study (17–22).

The TI-RADS from the ACR has been used since 2017 for the evaluation of thyroid nodules (16), which improves the diagnostic accuracy rate of thyroid nodules and has high rates of specificity (23–25). According to the ACR TI-RADS, thyroid nodules have multiple malignant features, and the assignment of different malignant features varies depending on their malignant potential. For example, if the peripheral(rim) calcifications are 2 points, and the extra-thyroidal extension is 3 points. Thyroid nodules with more suspicious features are given additional points. Those with a score greater than 7 points are classified as TR 5 and are likely to be more than 20% malignant. We consider that a higher score implies a higher malignant potential and a greater susceptibility to cervical LNM. Our study indicates that patients with highest ACR score >9 are more likely to develop cervical LNM, and this threshold value (9 points) is 2 points higher than the lower threshold value (7 points) of TR5 nodules, indicating that they have more suspicious features, have higher malignant potential, and have an increased risk of cervical LNM. This study also found that total ACR scores >19 points is associated with the risk of cervical LNM, which means that when there are more than 3 TR4 nodules in a case, the risk of cervical LNM increases. And there may also be another situation. According to ACR TI-RADS, a thyroid nodule can be rated as a maximum of 17 points. When there is a 17-point nodule in a case, adding any TR4 or TR5 nodule significantly increases the risk of cervical LNM. In summary, when the cumulative number of nodules or the cumulative suspicious features of the nodules reach the total ACR score >19, the patient has a greater susceptibility of cervical LNM.

No consensus has been established regarding whether the TTD (sum of the maximum diameter of each cancer nodule) is correlated with the incidence of central lymph node metastasis (CLNM) in PTMC (26, 27). Relevant studies have all used 10 mm as the cutoff value of TTD (28, 29). In our study, patients with multifocal cancer were divided into two groups based on whether the TTD was greater than 8 mm. This basis was selected by comparing the Youden index. The results showed no significant difference in LNM between the TTD > 0.8 cm group and TTD <0.8 cm group (P>0.005).Interestingly, in the univariate analysis, the number of thyroid nodules, number of thyroid lobes occupied by thyroid nodules and TTD >0.8 cm were significantly associated with LN status. However, they were not included in the final nomogram. We found that the strong discriminatory power of the ACR score diminished the value of those three factors in the final multivariate logistic regression analysis. The TTD is the sum of the maximum diameters of all nodules in a single case, and we suggest that it is correlated with the number of nodules in a single case. In our study, the number of nodules was also not an independent risk factor, which may explain the inconsistency between the results of this study and those of other studies. The results of this study may have occurred for the following reasons. (1) In this study, mPTMC with a TTD >8 mm had a relatively low composition ratio in the total mPTMC population (56.1%). The proportion of TTD >10 mm was only 37.9%, which was significantly lower than that in similar studies (26, 27, 30, 31). (2) In this study, after removing the primary nodules, only 9.5% of the secondary nodules had a maximum diameter >5 mm, and the remaining lesions were all <5 mm. Luo (20) suggested that cancer nodules with a maximum diameter of less than 5 mm were less likely to show enlargement or enhanced invasiveness, and related data also indicated that the incidence of CLNM was lower for cancer nodules with small diameters, which may also be one reason why no significant difference in CLNM was found between this patient type and patients with unifocal cancer.

This study shows that the number of nodules is a risk factor for LNM (P=0.083), but not an independent risk factor (P>0.05), indicating that it is associated with cervical LNM, but not an independent predictor, which is inconsistency with same type research. There are two main reasons, on the one hand, the relevant research did not separate unifocal PTMC (uPTMC) and mPTMC. However, this study excluded uPTMC and the only research object was mPTMC, which to some extent weakened the impact of the number of nodules. Another reason is that this study included features such as the total ACR scores, which is related to the number of nodules. For example, in case A, there are five PTMCs with highest ACR score of 7 points, 6 points, 7 points, 7 points, and 6 points, respectively, with a total ACR scores of 33 points; Case B has two PTMCs with highest ACR scores of 17 points and 16 points, respectively, and its total ACR scores is 33 points. According to the results of this study, the risk of LNM in both cases is consistent, but the number of lesions in case B is smaller than that in case A. The total ACR scores have weakened the impact of the number of nodules in the final model, leading to inconsistencies between the results of this study and other studies (17–22).

Our study developed and validated an ultrasound radiomics nomogram that had good accuracy and consistency, possibly because we reviewed a large amount of literature and selected risk factors that may be associated with the occurrence of LNM in mPTMC for the analysis, as much as possible, and because we constructed ROC curves for the risk factors and used the cutoff values as the basis for grouping each factor. For example, TTD was divided into >8 mm and ≤8 mm groups instead of using the 10 mm grouping criterion to avoid affecting the results of the subsequent regression analysis due to the uneven distribution of data between groups. Our nomogram can predict the risk of cervical LNM through basic patient information and ultrasound features before surgery, providing guidance for clinical treatment decisions. High risk patients receive early surgical treatment to obtain a good prognosis, while low risk patients can undergo active detection to avoid overtreatment.

Our study is innovative in several regards. First, our study distinguishes multifocal from unifocal cases. Second, our study is the first to examine mPTMC and to predict the occurrence of LNM in mPTMC. Moreover, our study introduced the ACR TI-RADS score as a risk factor.

The limitations of this study include the small number of included cases and its single-center design. The predictive performance of this model requires verification in studies with a large number of multicenter cases.

## 5 Conclusion

This study puts forward a radiomics nomogram, which is based on ACR TI-RADS scores, shows favorable predictive value for the preoperative assessment of LNs in patients with mPTMC. These findings may provide a basis for surgical decision making and the extent of tumor resection.

## Data availability statement

The raw data supporting the conclusions of this article will be made available by the authors, without undue reservation.

## Ethics statement

The studies involving human participants were reviewed and approved by Ethics Committee of the China-Japan Union Hospital of Jilin University. The patients/participants provided their written informed consent to participate in this study. Written informed consent was obtained from the individual(s) for the publication of any potentially identifiable images or data included in this article.

## Author contributions

All authors listed have made a substantial, direct and intellectual contribution to the work, and approved it for publication.

## References

[1] Al Afif A Williams BA Rigby MH Bullock MJ Taylor SM Trites J. Multifocal papillary thyroid cancer increases the risk of central lymph node metastasis. Thyroid (2015) 25(9):1008–12. doi: 10.1089/thy.2015.0130 26161997

[2] Alabousi M Alabousi A Adham S Pozdnyakov A Ramadan S Chaudhari H. Diagnostic test accuracy of ultrasonography vs computed tomography for papillary thyroid cancer cervical lymph node metastasis: a systematic review and meta-analysis. JAMA otolaryngology– Head Neck Surg (2022) 148(2):107–18. doi: 10.1001/jamaoto.2021.3387 PMC8613701 34817554

[3] Joseph KR Edirimanne S Eslick GD. Multifocality as a prognostic factor in thyroid cancer: a meta-analysis. Int J Surg (London England) (2018) 50:121–5. doi: 10.1016/j.ijsu.2017.12.035 29337178

[4] Nagaoka R Ebina A Toda K Jikuzono T Saitou M Sen M. Multifocality and progression of papillary thyroid microcarcinoma during active surveillance. World J Surg (2021) 45(9):2769–76. doi: 10.1007/s00268-021-06185-2 34100116

[5] Kim KJ Kim SM Lee YS Chung WY Chang HS Park CS. Prognostic significance of tumor multifocality in papillary thyroid carcinoma and its relationship with primary tumor size: a retrospective study of 2,309 consecutive patients. Ann Surg Oncol (2015) 22(1):125–31. doi: 10.1245/s10434-014-3899-8 25092159

[6] Sugitani I Ito Y Takeuchi D Nakayama H Masaki C Shindo H. Indications and strategy for active surveillance of adult low-risk papillary thyroid microcarcinoma: consensus statements from the Japan association of endocrine surgery task force on management for papillary thyroid microcarcinoma. Thyroid (2021) 31(2):183–92. doi: 10.1089/thy.2020.0330 PMC7891203 33023426

[7] Zhao WJ Luo H Zhou YM Dai WY Zhu JQ. Evaluating the effectiveness of prophylactic central neck dissection with total thyroidectomy for cN0 papillary thyroid carcinoma: an updated meta-analysis. Eur J Surg Oncol J Eur Soc Surg Oncol Br Assoc Surg Oncol (2017) 43(11):1989–2000. doi: 10.1016/j.ejso.2017.07.008 28807633

[8] Chen L Wu YH Lee CH Chen HA Loh EW Tam KW. Prophylactic central neck dissection for papillary thyroid carcinoma with clinically uninvolved central neck lymph nodes: a systematic review and meta-analysis. World J Surg (2018) 42(9):2846–57. doi: 10.1007/s00268-018-4547-4 29488066

[9] Zhao W You L Hou X Chen S Ren X Chen G. The effect of prophylactic central neck dissection on locoregional recurrence in papillary thyroid cancer after total thyroidectomy: a systematic review and meta-analysis: pCND for the locoregional recurrence of papillary thyroid cancer. Ann Surg Oncol (2017) 24(8):2189–98. doi: 10.1245/s10434-016-5691-4 27913945

[10] Dobrinja C Troian M Cipolat Mis T Rebez G Bernardi S Fabris B. Rationality in prophylactic central neck dissection in clinically node-negative (cN0) papillary thyroid carcinoma: is there anything more to say? a decade experience in a single-center. Int J Surg (London England) (2017) 41(Suppl 1):S40–s7. doi: 10.1016/j.ijsu.2017.01.113 28506412

[11] Haugen BR Alexander EK Bible KC Doherty GM Mandel SJ Nikiforov YE. 2015 American Thyroid association management guidelines for adult patients with thyroid nodules and differentiated thyroid cancer: the American thyroid association guidelines task force on thyroid nodules and differentiated thyroid cancer. Thyroid (2016) 26(1):1–133. doi: 10.1089/thy.2015.0020 26462967 PMC4739132

[12] Lee X Gao M Ji Y Yu Y Feng Y Li Y. Analysis of differential BRAF(V600E) mutational status in high aggressive papillary thyroid microcarcinoma. Ann Surg Oncol (2009) 16(2):240–5. doi: 10.1245/s10434-008-0233-3 19034577

[13] Mechanic RE Galvin RS. Self-insured employers - the payment-reform wild card. N Engl J Med (2018) 379(4):308–10. doi: 10.1056/NEJMp1801544 30044936

[14] Ahn JE Lee JH Yi JS Shong YK Hong SJ Lee DH. Diagnostic accuracy of CT and ultrasonography for evaluating metastatic cervical lymph nodes in patients with thyroid cancer. World J Surg (2008) 32(7):1552–8. doi: 10.1007/s00268-008-9588-7 18408961

[15] Hwang HS Orloff LA. Efficacy of preoperative neck ultrasound in the detection of cervical lymph node metastasis from thyroid cancer. Laryngoscope (2011) 121(3):487–91. doi: 10.1002/lary.21227 21344423

[16] Zhao L Sun X Luo Y Wang F Lyu Z. Clinical and pathologic predictors of lymph node metastasis in papillary thyroid microcarcinomas. Ann Diagn Pathol (2020) 49:151647. doi: 10.1016/j.anndiagpath.2020.151647 33126150

[17] Goran M Markovic I Buta M Gavrilovic D Cvetkovic A Santrac N. The influence of papillary thyroid microcarcinomas size on the occurrence of lymph node metastases. J BUON Off J Balkan Union Oncol (2019) 24(5):2120–6. 31786884

[18] Ito Y Miyauchi A Kihara M Higashiyama T Kobayashi K Miya A. Patient age is significantly related to the progression of papillary microcarcinoma of the thyroid under observation. Thyroid (2014) 24(1):27–34. doi: 10.1089/thy.2013.0367 24001104 PMC3887422

[19] Song J Yan T Qiu W Fan Y Yang Z. Clinical analysis of risk factors for cervical lymph node metastasis in papillary thyroid microcarcinoma: a retrospective study of 3686 patients. Cancer Manage Res (2020) 12:2523–30. doi: 10.2147/CMAR.S250163 PMC7153998 32308489

[20] Luo Y Zhao Y Chen K Shen J Shi J Lu S. Clinical analysis of cervical lymph node metastasis risk factors in patients with papillary thyroid microcarcinoma. J endocrinological Invest (2019) 42(2):227–36. doi: 10.1007/s40618-018-0908-y PMC6394766 29876836

[21] Huang Y Yin Y Zhou W. Risk factors for central and lateral lymph node metastases in patients with papillary thyroid micro-carcinoma: retrospective analysis on 484 cases. Front Endocrinol (2021) 12:640565. doi: 10.3389/fendo.2021.640565 PMC7973362 33746905

[22] Tessler FN Middleton WD Grant EG Teefey SA Abinanti N Boschini FJ. ACR thyroid imaging, reporting and data system (TI-RADS): white paper of the ACR TI-RADS committee. J Am Coll Radiol JACR (2017) 14(5):587–95. doi: 10.1016/j.jacr.2017.01.046 28372962

[23] Griffin AS Mitsky J Rawal U Bronner AJ Tessler FN Hoang JK. Improved quality of thyroid ultrasound reports after implementation of the ACR thyroid imaging reporting and data system nodule lexicon and risk stratification system. J Am Coll Radiol JACR (2018) 15(5):743–8. doi: 10.1016/j.jacr.2018.01.024 29503150

[24] Hoang JK Middleton WD Farjat AE Teefey SA Abinanti N Boschini FJ. Interobserver variability of sonographic features used in the American college of radiology thyroid imaging reporting and data system. AJR Am J Roentgenol (2018) 211(1):162–7. doi: 10.2214/AJR.17.19192 29702015

[25] Ha EJ Na DG Baek JH Sung JY Kim JH Kang SY. US Fine-needle aspiration biopsy for thyroid malignancy: diagnostic performance of seven society guidelines applied to 2000 thyroid nodules. Radiology (2018) 287(3):893–900. doi: 10.1148/radiol.2018171074 29465333

[26] Zhao Q Ming J Liu C Shi L Xu X Nie X. Multifocality and total tumor diameter predict central neck lymph node metastases in papillary thyroid microcarcinoma. Ann Surg Oncol (2013) 20(3):746–52. doi: 10.1245/s10434-012-2654-2 22972508

[27] Liu C Wang S Zeng W Guo Y Liu Z Huang T. Total tumour diameter is superior to unifocal diameter as a predictor of papillary thyroid microcarcinoma prognosis. Sci Rep (2017) 7(1):1846. doi: 10.1038/s41598-017-02165-6 28500312 PMC5431972

[28] Feng JW Pan H Wang L Ye J Jiang Y Qu Z. Total tumor diameter: the neglected value in papillary thyroid microcarcinoma. J Endocrinol Invest (2020) 43(5):601–13. doi: 10.1007/s40618-019-01147-x 31749082

[29] Tam AA Ozdemir D Ogmen BE Fakı S Dumlu EG Yazgan AK. Should multifocal papillary thyroid carcinomas classified as t1a with a tumor diameter sum of 1 to 2 centimeters be reclassified as T1B? Endocr Pract (2017) 23(5):526–35. doi: 10.4158/EP161488.OR 28156153

[30] Jiang KC Lin B Zhang Y Zhao LQ Luo DC. Total tumor diameter is a better indicator of multifocal papillary thyroid microcarcinoma: a propensity score matching analysis. Front Endocrinol (2022) 13:974755. doi: 10.3389/fendo.2022.974755 PMC9393720 36004348

[31] Hîțu L Ștefan PA Piciu D. Total tumor diameter and unilateral multifocality as independent predictor factors for metastatic papillary thyroid microcarcinoma. J Clin Med (2021) 10(16):3707. doi: 10.3390/jcm10163707 PMC8396836 34442001

